# SCREENING FOR MENTAL HEALTH PROBLEMS IN PRESCHOOLERS AT PRIMARY HEALTH CARE SETTINGS

**DOI:** 10.1590/1984-0462/;2018;36;1;00009

**Published:** 2017-12-07

**Authors:** Raquel Godinho Hokama dos Santos, Eloisa Helena Rubelo Valler Celeri

**Affiliations:** a Universidade Estadual de Campinas, Campinas, SP, Brasil.

**Keywords:** Child, Preschool, Mental health, Primary health care, Pré-escolar, Saúde mental, Atenção primária à saúde

## Abstract

**Objective:**

To study the applicability of the *Strength and Difficulties Questionnaire* (SDQ 2,4-p) as a screening tool for mental health problems in preschoolers, in the context of Primary Health Care; to evaluate the mental health problems of the sample, comparing data from SDQ (2,4-p) and from the *Child Behavior Check List* (CBCL 1½-5 years).

**Methods:**

Cross-sectional observational study with a convenience sample of 31-50-month-old children, whose caretakers provided informational reports. In the first stage, professionals from the primary care health unit have applied the SDQ (2,4-p) during routine appointments. Subsequently, the CBCL (1½-5) was applied by a professional experienced in infant mental health. The SDQ and CBCL results were compared and the correlation between the scales was tested.

**Results:**

Among 280 questionnaires available to the health professionals, 48 were filled out and the CBCL was applied to 40 of the participants. Among the problems found with the SDQ, 18 cases (37.6% out of 48) have shown abnormal score in the “Total Difficulties” and 38 (80.9% out of 48) have shown normal score in the “Impact of Difficulty”. Behavioral issues were highlighted by the percentage of abnormal scores (47.9%). The correlation between SQD and CBCL was positive for all scales, except for the pro-social behavior.

**Conclusions:**

Clinically important mental health problems were found in preschool children. Variables of the SDQ discriminate normal and abnormal scores according to the CBCL parameters, thus functioning as a good screening tool.

## RESUMO

**Objetivo:**

Estudar a aplicabilidade do Questionário de Capacidades e Dificuldades - *Strength and Difficulties Questionnaire* (SDQ 2,4-p) - como instrumento de rastreamento, na Atenção Básica à Saúde (ABS), de problemas de saúde mental em crianças pré-escolares; caracterizar os problemas de saúde mental da amostra, comparando dados do SDQ (2,4-p) e do Inventário de Comportamentos da Criança - *Child Behavior Check List* (CBCL 1½-5 anos).

**Métodos:**

Estudo observacional transversal com amostra de conveniência composta por crianças de 31 a 50 meses, cujos responsáveis forneceram relato das informações. Na primeira etapa, profissionais da unidade básica de saúde (UBS) aplicaram o SDQ (2,4-p) durante consultas de rotina. Na sequência, o CBCL (1½-5) foi aplicado aos responsáveis por profissional experiente especializado em saúde mental infantil. Resultados do SDQ e do CBCL foram comparados; correlação entre as escalas foi analisada.

**Resultados:**

Dos 280 questionários disponibilizados aos profissionais da UBS, 48 foram preenchidos e houve aplicação do CBCL em 40 dos participantes. Entre os problemas rastreados com o SDQ, 18 casos (37,6% de 48) apresentam escore anormal no “Total de Dificuldades” e 38 (80,9% de 48), escore normal no “Impacto da Dificuldade”. Problemas de conduta se destacaram pela porcentagem de escores anormais (47,9%). A correlação entre SDQ e CBCL foi positiva em todas as escalas, exceto no comportamento pró-social.

**Conclusões:**

Problemas de saúde mental clinicamente importantes foram encontrados em crianças pré-escolares. Variáveis do SDQ discriminam escores normais e anormais, conforme parâmetros do CBCL, funcionando como um bom instrumento de triagem.

## INTRODUCTION

The early years of a child’s life are decisive for his or her development and health. The neural circuits are rapidly being established, thus being influenced by early experiences, which impact infant subjectivity, neural organization and behavior, in the same proportion as the innate characteristics of the children and/or of the environment in which development takes place.^1^

Mental health problems (MHPs) interfere in the quality of early experiences, and, therefore, in the development of the children’s skills. They have a negative impact on infant adaptation to environmental demands, acquisition of new abilities and capacities, as well as interpersonal functioning, and the father-mother-child relationship.^2^ They may not produce stable symptoms, manifesting in different forms, according to each stage of development, which makes them difficult to identify.^3^

Despite the popular belief that MHPs resolve themselves once the child grows up, they are specially lasting when they appear at that stage.^4^ Persistence is more common if the MHPs are present in more than one developmental domain, or when the parents complain about the impact on family routine.^5^ In spite of that, only a small group of children with clinical MHP is identified and treated in health services.^6^

Primary Health Care (PHC) is a privileged aspect of the Health Care Networks composing the Brazilian public health system to detect this infant demand. This care environment provides the longitudinal follow-up of the children, promoting whole health care and contextualizing factors that determine the quality of life and health of the children, in relation to their community.^7^ ^,^ ^8^ ^,^ ^9^ As a paradox, many professionals who work in BHC do not feel capable and comfortable to identify and handle possible infant MHPs.^10^ The use of standardized screening instruments of MHPs in young children can be a strategy for these challenges. Especially in PHC, they can help identify the more severe cases, which require close and/or specialized follow-up, thus contributing with the elaboration of effective therapeutic projects.^11^

Considering the shortage of studies about strategies of qualification to detect MHP in the Brazilian PHC, this study aimed at: analyzing the applicability of the *Strength and Difficulties Questionnaire* (SDQ 2,4-p) as an instrument to identify MHPs in preschoolers, in the context of BHC, and to characterize the MHPs in the sample analyzed by comparing the data in SDQ (2,4-p) with those in the *Child Behavior Check List* (CBCL 1½-5 years).

## METHOD

This study was approved by the Research Ethics Committee of the School of Medical Sciences at Universidade Estadual de Campinas (n^.^ 47843315.1.0000.5404), and the informed consent form was signed during the first stage of collection.

This is a cross-sectional, observational study. The convenience sample was composed of users of a primary health care unit (PHCU), located in a city of a metropolitan region in the countryside of the state of São Paulo.

The PHCU for the study was selected by stratification, and the selection criteria included:

- Territories with a larger population of children aged between zero and four years old, excluding those in which the socioeconomic vulnerability of the population was the prevalent condition;
- PHCU with more complete professional team; and
- Demand for care more compatible with the possibility of the service.

The evaluation of PHCUs was conducted by a representative in the management team of the municipal administration.

Of the users in the PHCU selected, the following were considered as research subjects: children aged from 30 to 50 months, assisted in the period of September 2015 to June 2016, without exclusion criteria. The data were collected by the report of primary caretakers.

The following measurement instruments were used:

- Brazilian Criteria of Economic Classification (CCEB);
- Strengths and Difficulties Questionnaire - SDQ); e
- Child Behavior Check List *-* CBCL 1 ½-5).

The CCEB was elaborated by the Brazilian Association of Research Companies (ABEP) to identify the consumption potential of Brazilian families. The items assessed are:

- Possession of durable consumer goods
- Type of water supply system and street paving;
- Number of people living in the household;
- Family composition; and
- Schooling of the head of the family.

It stratifies the population in the categories: A1, A2, B1, B2, C1, C2, D and E.^12^

SDQ is a screening instrument for MHPs created by Robert Goodman in the 1990s.^13^ Since then, it has been widely used in scientific research and in the clinical context to identify MHPs in children and adolescents, and to assess the severity of symptoms and/or the impact of psychopathology.^14^

Its broad use is owed to factors such as good acceptance by the informers, facility to score in the scales, effectiveness in the detection of MHPs and consideration of the children’s competences.^15^ There are versions in different languages for ages between two and 17, applicable to parents and teachers, besides a self-applicable version for children aged more than 11. In Brazil, the translation and validation of this instrument were carried out by Fleitlich-Bylik et al., in 2000.^15^

The structure of SDQ is composed of three clusters, called “psychological attributes”, “impact supplement”, and “follow-up”. The “psychological attributes” contain 25 items that assess five subscales: emotional symptoms, conduct problems, hyperactivity, peer relationship problems, and prosocial behavior.^13^ Of the five subscales, four screen behaviors associated with problems and, together, “provide the total difficulties score” of the child. The fifth deals with one competence, the “prosocial behavior”. For each evaluation, the “normal”, “borderline” or “abnormal classifications were presented.^13^ The “impact supplement” points to the chronicity of symptoms and the impact of the difficulty on the child and his or her daily routine and family life. Finally, the “follow-up” identifies symptomatic changes throughout time, as a response to therapeutic interventions.^13^

This paper used the “psychological attributes” and the “impact supplement”, from the version two to four years for parents (SDQ 2,4-p), available for free use.^16^ The classification of scores was in accordance with SDQ’s normative, facing the absence of a cutoff reference for the Brazilian population of Brazilian preschoolers.

The CBCL is an instrument used to assess socioemotional and behavioral problems created by Thomas Achenbach in the 1960s. Such instrument is part of a set of inventories called The Achenbach System of Empirically Based Assessment (ASEBA).^17^ The version for children aged between 1½ and 5 years is composed of 99 items to be answered by the primary caretakers of the children assessed. The items evaluate seven “syndrome scales”:

1. “Emotionally reactive”;
2. “Anxious-depressed”;
3. “Somatic complaints”;
4. “Withdrawn”;
5. “Sleep problems”;
6. “Attention-hyperactivity problems”; and
7. “Aggressive behavior”.

The first four syndrome scales are grouped and constitute the “total internalizing problems”, the last two form the “total externalizing problems”. The seven syndrome scales, together, express the “total emotional and behavioral problems” (TP). Each evaluation is classified as “normal”, “borderline” or “clinical”. Besides the result indicating the syndrome scales, CBCL also assesses “stress problems” generates profiled oriented by the Diagnostic and Statistical Manual of Mental Disorders - IV (DSM-IV).^17^

The adaptation of CBCL ½-5 for the Brazilian culture it not validated yet. However, a study by Ivanova points to the possibility of generalizing the model of the seven syndrome scales, based on the conclusion that the instrument captures socioemotional and behavioral problems reported by parents with cultural experiences that are very diverse.^18^ CBCL is widely used in studies of child and adolescence socioemotional and behavioral assessment, standing out for its effectiveness.^11^ In Brazil, it is distributed by the team of ASEBA Brazil, and it is necessary to purchase the issues and the software of systematization and data score.

**Figura 1:**
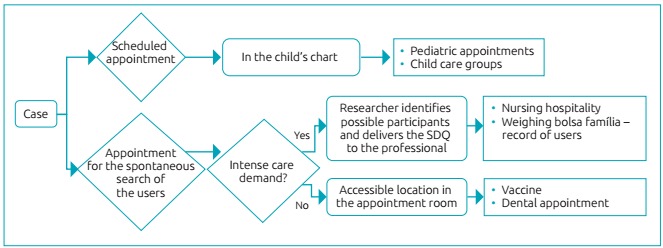
Flowchart of the distribuion in SDQ queries.

Before data collection, we organized meetings with health professionals from PHCUs, in order to present the research and the SDQ questionnaire, list the people interested in collaborating with the study and elaborate strategies for collection, adjusting the research procedures to the routine of the professionals. The procedures consisted of two stages with an interval shorter than 30 days. In the first one, SDQ was applied by a PHCU professional, during the appointment. In the collection period, the questionnaire was made available in all consultations of children whose profiles were compatible with the study. Figure 1 illustrates how the questionnaires were distributed to the professionals.

The collection data were systematized with the Statistical Analysis System^19^ (SAS System for Windows, 9.4). The sample was characterized by descriptive analysis, with measurements of frequency for qualitative variables, and of position and dispersion for quantitative ones. The borderline scores, understood as “risk for the development of MHP”, were added to the clinical scores. The linear association between the SDQ and CBCL variables was verified by the Spearman’s correlation coefficient. The intensity of the correlation was interpreted according to the values: 0.0 to 0.39 - mild; 0.4 to 0.59 - moderate; 0.6 to 1.0, strong intensity.^20^

To analyze the discriminatory capacity of the SDQ, the results of the “normal”, “borderline” and “abnormal” groups of the subscales were compared to the T scores (cutoff points) of the syndrome scales of CBCL using the Kruskal-Wallis test, followed by the Dunn test, to locate the differences between groups, when necessary.

The variables sex, age and socioeconomic class were compared to those in SDQ and CBCL using the chi-squared and Fisher’s exact tests. The significance level adopted in the tests was 5%.

## RESULTS

In the study period, 280 SDQ questionnaires were available for professionals in the PHCU. Of these, 48 were filled out, representing a rate of return of 17.1%. CBCL was applied in 40 of the 48 participants (83.3%); among the losses, one was owed to abandonment and seven were a result of out-of-date register data in the charts.

Of the 48 participants, 26 were female (54.2%). The ages ranged between 31 (n=3) and 50 months (n=1); the mean was 40 and the median was 39 months. There were mostly families with socioeconomic level C (n=22 - 55%), followed by 11 families (27.5%) in situation of social vulnerability (strata D and E), and 7 (17.5%) in stratum B. The study of the variables sx and socioeconomic level found statistically significant difference only in “sleep problems”: classes D and E: 56.9±8.3; class B: 53.4±4.0; and class C: 50.4±1.5 (p=0.004).

Table 1 presents the score of SDQ subscales. Normal scores appear more frequently in the subscale “prosocial behavior” and in the item “impact of the difficulty”. “Conduct problems”, “emotional symptoms” and “peer relationship problems” have higher prevalence of abnormal scores, among the five subscales considered. The item “total of difficulties” shows abnormal scores in 18 cases (37.5%), and borderline in 7 (14.6%), indicating that more than half of the children studied presented with risk for the development of MHPs.

**Table 1:** Score of the subscales in the Strength and Difficultlies Questionnaire - SDQ (n=48).

Subscales	M	SD	MD	NL	BDL	ANL
			(mín.-máx.)	n (%)	n (%)	n (%)
Conduct problems	4.6	±2.5	4 (0-10)	15 (31.3)	10 (20.8)	23 (47.9)
Emotional symptoms	2.9	±2.3	2.5 (0-9)	24 (50.0)	8 (16.7)	16 (33.3)
Peer relationship problems	2.5	±1.9	2 (0-6)	27 (56.3)	7 (14.6)	14 (29.2)
Hyperactivity problem	4.4	±3	5 (0-10)	29 (60.4)	13 (27.1)	6 (12.5)
Prosocial behavior	8.5	±1.7	9 (4-10)	40 (83.3)	4 (8.3)	4 (8.3)
Total of difficulties	14.4	±7.2	15 (2-27)	23 (47.9)	7 (14.6)	18 (37.5)
Impact of the difficulty	0.4	±1	0 (0-5)	38 (80.9)	4 (8.5)	5 (10.6)

M: mean; SD: standard deviation; MD: median; NL: normal; BDL: borderline ANL: abnormal.

Table 2 shows the score of the syndrome scales in CBCL. “Emotionally reactive” and “aggressive behavior” are the syndrome scales that most express clinical scores, and present the highest percentage of clinical and borderline scores, when added (17.5%).

**Table 2:** Score of the syndrome scales in the Child Behavior Check-List - CBCL 1 ½-5.

	M	SD	MD	NL	BDL	CLI
			(min.-max.)	n (%)	n (%)	n (%)
Emotionally reactive	56.3	±7.4	52 (56-76)	33 (82.5)	3 (7.5)	4 (10)
Anxiety/depression	54.6	±5.7	52 (50-69)	38 (95)	2 (5)	0
Somatic complaint	52.8	±4.1	51 (50-65)	39 (97.5)	1 (2.5)	0
Withdrawal	52.1	±4.1	51 (50-69)	39 (97.5)	1 (2.5)	0
Total internalizing problems	49.9	±9.8	51 (28-69)	34 (85)	4 (10)	2 (5)
Aggressive behavior	55.4	±7.1	52 (50-73)	33 (82.5)	4 (10)	3 (7.5)
Attention/hyperactivity	54.4	±5.9	51 (50-69)	35 (87.5)	5 (12.5)	0
Total externalizing problems	51	±11.4	52.5 (28-75)	32 (80)	1 (2.5)	7 (17.5)
Sleep problems	52.6	±5.4	50 (50-71)	37 (92.5)	2 (5)	1 (2.5)
Stress problems	55.2	±5.4	53 (50-68)	39 (97.5)	1 (2.5)	0
Total of problems	48.5	±10	48 (28-69)	34 (85%)	4 (10)	2 (5)

M: mean; SD: standard deviation; MD: median; min.: minimum value; max.: maximum value; n: number of subjects; NL: normal; BDL: borderline; CLI: clinical.

The item “total internalizing problems” has lower frequency of abnormal scores than the “total externalizing problems”, even by adding the clinical and borderline scores: 15 to 20%, respectively. The change in “total problems” reaches 15%.

Table 3 shows the statistically significant correlations and of moderate to strong intensity between the variables of the instruments. The correlations were positive between all the variables. One exception is established in the “prosocial behavior”, for having a statistically significant and negative correlation with “withdrawal” and “aggressive behavior”: the higher the “prosocial behavior” score, the lower the “aggressive behavior” score, which is one of the most common problems in the sample.

**Tabela 3:** Correlações estatisticamente significantes, moderadas a fortes, entre os escores das variáveis do SDQ e do CBCL (n=40).

SDQ Variables	CBCL Variables	r_**s**_	p-value
Emotional symptoms	Anxiety/depression	0.480	0.002
	Total internalizing problems	0.503	0.001
	Attention/hyperactivity	0.412	0.008
	Stress problem	0.546	<0.001
	Total of EB problems	0.437	0.005
	Anxiety/depression	0.437	0.005
Conduct problems	Total internalizing problems	0.501	0.001
	Aggressive behavior	0.600	<0.001
	Attention/hyperactivity	0.489	0.001
	Total externalizing problems	0.675	<0.001
	Stress problem	0.637	<0.001
	Total of EB problems	0.664	<0.001
Hyperactivity problem	Attention/hyperactivity	0.586	<0.001
	Stress problem	0.466	0.002
	Total of probelms	0.401	0.010
Peer relationship problem	Anxiety/depression	0.468	0.002
	Emotionally reactive	0.422	0.007
	Total internalizing problems	0.474	0.002
	Total of EB problems	0.440	0.004
Prosocial behavior	Aggressive behavior	-0.340	0.030
	Withdrawal	-0.330	0.036
Total of difficulty	Attention/hyperactivity	0.606	<0.001
	Aggressive behavior	0.486	0.001
	Total externalizing problems	0.595	<0.001
	Anxiety/depression	0.579	0.001
	Emotionally reactive	0.486	0.002
	Total internalizing problems	0.596	<0.001
	Stress problem	0.669	<0.001
	Total of EB problems	0.641	0.001
Impact of the difficulty	Aggressive behavior	0.540	<0.001
	Total externalizing EB problems	0.522	<0.001
	Total of problems	0.467	0.003

SDQ: *Strengths and Difficulties Questionnaire*; CBCL: *Child Behavior Check List*; *r*_*s*_: Spearman’s correlation coefficient; EB: emotional and behavioral.

Among other clinical problems often found, the “emotional symptoms” showed significant correlations of mild intensity with “emotionally reactive”, and moderate with the “total internalizing problems” and “total emotional and behavioral problems”. The “conduct problems” presented significant correlations of moderate to strong intensity with the “aggressive behavior”, “total externalizing problems” and “total emotional and behavioral problems”.

Table 4 presents the comparison between the T score of CBCL and the groups “normal”, “borderline” and “abnormal” only in the variables of SDQ which presented moderate to strong correlations with those in CBCL. There is a statistically significant difference between the “normal” and “abnormal” groups in almost all SDQ subscales.

**Table 4:** Differences between the normal, borderline, and abnormal groups in the SDQ variables in comparison to CBCL variables.

CBCL	SDQ	SDQ - ANL n, mean, SD	SDQ - BDL n, mean, SD	SDQ - NL n, mean, SD	p-value	Location of the difference
Emotionally reactive	Total of difficulties	14; 59.9; 8.7	5; 59.0; 9.6	21; 53.2; 4.4	0.022	ANL and NL
	Peer relationship	11; 60.9; 8.1	5; 53.2; 2.7	24; 54.8; 7.0	0.046	ANL and NL
Anxiety-depression	Total of difficulties	14; 58.9; 6.5	5; 54.2; 3.4	21; 51.7; 3.2	0.007	ANL and NL
	Emotional	13; 58.9; 6.5	7; 53.4; 4.6	20; 52.1; 3.5	0.010	ANL and NL
	Conduct	17; 57.4; 5.6	9; 52.9; 4.7	14; 52.2; 5.0	0.009	ANL and NL
	Peer relationship	11; 57.8; 5.4	5; 52.8; 3.6	24; 53.4; 5.7	0.039	ANL and NL
Withdrawal	Prosocial behavior	3; 54.7; 3.5	2; 51.0; 0.0	35; 51.9; 4.1	0.450	-
Total internalizing	Total of difficulties	14; 56.6; 6.4	5; 49.6; 12.8	21; 45.6; 8.7	0.003	ANL and NL
	Emotional	13; 56.3; 8.4	7; 48.6; 12.6	20; 46.3; 7.6	0.008	ANL and NL
	Conduct	17; 55.6; 6.4	9; 46.6; 11.1	14;45.1; 9.2	0.004	ANL and NL
	Peer relationship	11; 55.5; 6.5	5; 47.2; 6.5	24; 47.9; 10.7	0.025	ANL and NL
Attention-hyperactivity	Total of difficulties	14;58.0; 7.1	5; 54.0; 4.6	21; 52.0; 4.0	0.003	ANL and NL
	Emotional	13; 57.5; 7.0	7; 52.1; 4.1	20; 53.2; 4.9	0.020	ANL and NL
	Conduct	17; 56.6; 6.7	9; 52.8; 4.0	14; 52.7; 5.3	0.015	ANL and NL
	Hyperactivity	5; 58.4; 7.4	9; 57.7; 7.4	26; 52.5; 4.1	0.016	BDL and NL
Aggressive behavior	Total of difficulties	14; 58.7; 8.9	5; 52.6; 3.7	21; 53.9; 5.6	0.160	-
	Conduct	17; 59.9; 8.1	9; 53.1; 5.4	14; 51.4; 2.3	0.002	ANL and NL
	Prosocial behavior	3; 59.7; 9.3	2; 62.5; 14.8	35; 54.6; 6.4	0.180	-
	Impact of the difficulty	4; 61.5; 7.7	3; 68.3; 8.1	32; 53.4; 5.3	0.0027	ANL and NL
Total externalizing	Total of difficulties	14; 57.8; 10.1	5; 46.4; 11.7	21; 47.6; 11.1	0.034	ANL and NL
	Conduct	17;55.5; 9.1	9; 48.7; 10.4	14; 43.1; 9.7	<0.001	ANL and NL
	Impact of the difficulty	4; 62.5; 9.5	3; 65.3; 7.2	32; 48.0; 10.6	0.043	ANL and NL
Stress problem	Total of difficulties	14; 59.4; 5.6	5; 52.8; 2.7	21; 53.0; 4.1	0.006	ANL and NL
	Emotional	13; 59.9; 5.5	7; 54.7; 4.9	20; 52.3; 3.0	0.003	ANL and NL
	Conduct	17; 59.4; 5.0	9; 53.0; 4.1	14; 51.5; 2.5	<0.001	ANL and NL; ANL and BDL
	Hyperactivity	5; 59.2; 5.9	9; 57.9; 5.9	26; 53.5; 4.3	0.066	-
Total of problems	Emotional	13; 55.1; 8.1	7; 46.4; 10.5	20; 44.9; 9.2	0.015	ANL and NL
	Conduct	17; 55.5; 7.7	9; 45.8; 9.5	14; 41.7; 7.3	<0.001	ANL and NL
	Hyperactivity	5; 53.8; 10.9	9; 53.9; 11.8	26; 45.6; 8.2	0.032	BDL and NL
	Peer relationship	11; 55.5; 8.7	5; 44.0; 5.4	24; 46.2; 9.9	0.018	ANL and NL; ANL and BDL
	Impact of the difficulty	4; 54.8; 6.2	3; 63.0; 6.0	32; 46.0; 9.2	0.008	BDL and NL

CBCL: *Child Behavior Check List*; SDQ: *Strengths and Difficulties Questionnaire*; n: number of subjects; M: mean; SD: standard deviation; a: Kruskal-Wallis test; b: Dunn’s test; ANL: abnormal; BDL: borderline; NL: normal.

The item “total of difficulties” did not present a discriminatory capacity between “normal”, “borderline” and “abnormal” in comparison to “aggressive behavior”, in spite of comparing it to the other CBCL variables. Another variable with great percentage of abnormal scores, the “peer relationship”, distinguishes the groups “normal and abnormal”, “abnormal and borderline”, in comparison to the “total emotional and behavioral problems”, also distinguishing “normal and abnormal” in relation to “emotionally reactive”. The “impact of the difficulty” can discriminate the groups “normal and borderline”, in comparison to the “total of problems”.

## DISCUSSION

The main finding in this study refers to the capacity of SDQ to discriminate the groups of children in the sample, with normal and abnormal scores, in comparison to the evaluation obtained by CBCL. Besides, the instrument scales showed significant and positive correlations, suggesting the interdependence between the different aspects of socioemotional development in the young children.

“Aggressive behavior” and “tota externalizing problems” were the variables with the highest prevalence of clinical score in this study sample. Such results are similar to those observed in international and national studies involving preschoolers.^21^ ^,^ ^22^ ^,^ ^23^ ^,^ ^24^ Matijasevich et al. found, in Brazilian preschoolers, in a period of 11 years, a 25% increase in “total externalizing problems”, and 23.3% in “aggressive behavior”.^24^

In this study, the syndrome scale “aggressive behavior” presents a negative correlation with the subscale “prosocial behavior”, and the latter presents 83.3% of normal scores. It is possible to understand that, in the sample studied, the infant competence “prosocial behavior” may function as an important protective factor, supporting the resilience or even softening the impact of the behavioral problem of the child on his or her environment.

The difference between the variable sex and MHPs was not significant in this study, as it was not in a study conducted by Bao.^25^ The “sleep problem” had higher score in classes D and E, which may be related with the characteristics of the family households that composed the sample, such as the existence of a single sleeping room for all the members, possibly affecting the sleep routine of the child.^26^

The psychometric characteristics of the SDQ version for preschoolers have been examined in international studies, showing higher sensitivity of the instrument in relation to its specificity.^27^ ^,^ ^28^ This characteristic is expressed in this study by the discriminatory capacity, especially of the “normal” and “abnormal” scores, and by the compatibility between SDQ and CBCL, especially in the identification of “abnormal - clinical” problems. The compatibility is reinforced in cases in which the “total of difficulties” and “impact of the difficulty” present high scores.^28^

SDQ, as well as other standardized infant MHP screening instruments, is easy and fast to apply and score. Its use is flexible (it can be done in person, by phone or others), besides being free.^29^ These factors favor the use of SDQ as a measurement instrument in this study.

The use of standardized instruments in the MHP screening of preschoolers may facilitate the report of caretakers, once many have difficulties to communicate their concerns or fears, even when the socioemotional and behavioral difficulties of the children are clinically relevant.^30^ When the caretakers report their concerns to a professional, by a non-structured interview, their complaint is usually minimized.^29^ This reinforces the fact that, also for the health professional, the standardized instrument may help identify the problems, reducing the biases.

In this study, all informants were the primary caretakers of the children. The main advantage of this quality of informants are the appropriation over the development of the child, the context and the history of his or her behavior and temper, so it is possible to describe how the difficulty appears, manifests and changes with time.^29^ Simultaneously, it is important to consider there may be dissent between the perception of different caretakers about the difficulties and skills of the children, the expectations about their performance and their constitutional characteristics.^31^ This divergence in the relational environment may be exteriorized as reports of complaints from the caretakers about the child.

Once these factors are not measured in this study, the “abnormal (clinic” and “borderline”) results cannot be immediately interpreted as problems inherent to the child. Widely, such results indicate that something is affecting their whole socioemotional development, and point to the need to investigate MHPs in relation to the ecological context of infant development. Therefore, it is possible to prevent a medical and potentially iatrogenic approach for the suffering of the small child and his or her family.

The borderline scores were assumed as a risk indicator for the development of MHPs, therefore standing out from the “normal” scores. The objective is not to pathologize a slight deflection of a typical development path, but instead, to emphasize that the children with this score, as well as those with “abnormal-clinical” score, could be followed-up more closely by the BHC teams. The borderline score may function as a marker, showing that therapeutic efforts should be made with the child and his or her family to prevent more severe symptoms and promote the health infant development. In the Brazilian BHC context, these efforts may be owed to the articulation of the intra and inter-sectoral care network, to the discussion of cases in support meetings and/or to the implied referral, when necessary.^9^

The sampling size was the main limiting factor of the comprehension of this study, possibly because of the low adherence of BHU professionals, which occurred in spite of the procedures adopted, which prioritized the non-overload of the work process. However, the findings encourage and point to the need to explore the early intervention field for MHPs in the interface with the Brazilian public health. 

It is possible to conclude that preschoolers may present with clinically important MHPs. SDQ was effective in the identification of internalizing and externalizing problems, functioning as a good screening instrument. Given this feature, it may assist PHCU professionals to identify and monitor such problems, qualifying their therapeutic decisions.
